# Efficacy of fluticasone furoate nasal spray and levocetirizine in patients with Japanese cedar pollinosis subjected to an artificial exposure chamber

**DOI:** 10.3109/21556660.2013.829070

**Published:** 2013-07-26

**Authors:** Kazuhiro Hashiguchi, Sho Kanzaki, Ken-ichiro Wakabayashi, Nobuaki Tanaka, Kayoko Kawashima, Kiyochika Suematsu, Shoji Tokunaga, Kaoru Ogawa, Kimihiro Okubo

**Affiliations:** 1 Department of Otolaryngology, Futaba Clinic, TokyoJapan; 2 Medical Corporation Shinanokai, Samoncho Clinic, TokyoJapan; 3 Department of Otorhinolaryngology, Keio University School of Medicine, TokyoJapan; 4 Department of Otorhinolaryngology, Kitasato Institute Hospital, TokyoJapan; 5 Tanaka ENT Clinic, TokyoJapan; 6 Department of Otorhinolaryngology, Tokyo Women’s Medical University Medical Center East, TokyoJapan; 7 Department of Otorhinolaryngology, Otemae Hospital, OsakaJapan; 8 Medical Information Center, Kyushu University Hospital, FukuokaJapan; 9 Department of Otolaryngology, Nippon Medical School, TokyoJapan

**Keywords:** Japanese cedar pollinosis, Artificial exposure chamber, Combination therapy, Fluticasone furoate nasal spray, Levocetirizine

## Abstract

**Objective:**

This study investigated the clinical efficacy of a combination therapy of levocetirizine (LCTZ) and fluticasone furoate nasal spray (FFNS), compared with LCTZ monotherapy, for the suppression of seasonal allergic rhinitis (SAR) symptoms induced in an artificial exposure chamber.

**Methods:**

This study was a single-center, placebo-controlled, randomized, 3-way cross-over comparative study performed in 42 Japanese cedar pollinosis patients. These subjects received (1) LCTZ plus FFNS (combination group), (2) LCTZ plus FFNS placebo (monotherapy group), or (3) LCTZ placebo plus FFNS placebo (placebo group) once on the night prior to exposure, with a 1-week washout period between exposures. Nasal (sneezing, rhinorrhea, nasal congestion, and itchy nose) and ocular (eye itching and tearing) symptoms were recorded every 15 min, and the number of sneezes, nose blowing events, and the amount of nasal secretions were measured during exposure. The primary end-point was the cumulative incidence of SAR symptoms during exposure and the ‘ime to occurrence of symptoms’. The secondary end-points were the total nasal symptom score, the ocular symptom score, the amount of nasal discharge, and the number of sneezes and nose blowing events.

**Results:**

At all the measurement points, the lowest cumulative incidences for the nasal symptoms were observed in the combination group, followed by the monotherapy and placebo groups. All the subjects in the placebo group developed nasal symptoms within 2 h after pollen exposure, while three and eight subjects in the monotherapy and combination groups, respectively, did not develop any nasal symptoms during exposure. In addition, combination therapy delayed the onset of symptoms.

**Conclusions:**

The results demonstrated that combination therapy with FFNS and LCTZ significantly suppressed the induced SAR symptoms and delayed the onset of symptoms compared with LCTZ monotherapy and placebo. Although the conditions of the allergen challenge study using an exposure chamber are different from those in real life, combination therapy with FF and LCTZ was confirmed to be an effective treatment for SAR.

## Introduction

The prevalence of allergic rhinitis (AR) has increased both globally1 and in Japan2. The prevalence of seasonal allergic rhinitis (SAR) caused by Japanese cedar (JC) has increased over the past 10 years and is now estimated to affect up to 26% of the Japanese population, according to nationwide surveillance data2. Therefore, JC pollinosis has become a significant disease in Japan.

SAR symptoms, including rhinorrhea, sneezing attacks, nasal congestion, eye itching, and eye tearing, disturb not only daily life, but also socioeconomics3. These symptoms are known to have a negative impact on daily work, school performance, and quality-of-life3,4. Therefore, pollinosis patients are eager to receive appropriate medication against SAR symptoms.

According to the ARIA guidelines5, a second-generation H1-anti-histamine (AH) is recommended as the first line therapy in patients with mild-to-moderate symptoms, while intra-nasal corticosteroids (INSs) are recommended for patients with moderate-to-severe symptoms5. If such a treatment does not have a satisfactory effect, combination therapy with an AH or a leukotriene receptor antagonist (LTRA) is possible.

Systematic reviews have shown that INSs are superior to AHs or LTRAs for the suppression of symptoms of AR6,7. In addition, several clinical trials have shown that no clinical advantages exist for combination therapy of SAR, compared with INS monotherapy7–11. Although INSs could be considered as the most efficient drugs among several classes of medications for the treatment of AR, the proportion of prescriptions for AHs and combination therapy of AH and INS were both higher than that for INS monotherapy according to surveys conducted in the US12 and in Europe13.

The Japanese guidelines for AR (JP-MRG) discuss and recommend treatment plans for patients with JC pollinosis in consideration of the circumstances in Japan2. The strategy for treating pollinosis is described in detail according to disease severity and type (rhinorrhea and sneezing-dominant type and/or nasal congestion dominant type). For instance, the daily use of an INS is recommended in case of moderate/severe pollinosis with nasal congestion-dominant symptoms or with all three symptoms. If the efficacy is not sufficient, additional treatment with an AH and/or LTRA is recommended.

Many JC pollinosis patients consult not only ENT doctors, but also general physicians (GPs) or internal medicine doctors (IMs). Second-generation AHs were the most widely prescribed drugs according to a study investigating the prescription patterns for the treatment of JC pollinosis by ENT doctors, GPs, and IMs using a clinical vignette questionnaire14. Interestingly, GPs and IMs had a lower tendency to use INSs than ENT doctors, even if the patient’s symptoms became severe. In addition, an internet survey conducted by Konno and Kubo15 on the attitude of JC pollinosis patients toward drug adherence showed that 72% of the patients who were prescribed AHs (*n* = 8599) took the drug(s) regularly, just as prescribed, on the other hand only 38% of the patients who were prescribed INS (*n* = 4552) used the drug every day or frequently, while the remainder used the drug on demand. In this respect, INSs are not widely used in Japan because of the preferences of patients and a lack of understanding regarding their efficacy16. Therefore, the administration of second-generation AHs has been the mainstay of treatment for SAR, and the single use of INS presents difficulties, even if the patient’s symptoms are severe, in the present clinical setting in Japan.

Levocetirizine (LCTZ) is a second-generation AH, approved in Japan in 2009 for the treatment of perennial and seasonal allergic rhinitis. LCTZ has exhibited anti-histamine and several anti-inflammatory effects at clinically relevant concentrations in both *in vivo* and *in vitro* allergic studies17. A number of randomized, double-blind, placebo-controlled comparative studies have been published evaluating the efficacy of this drug for the treatment of SAR under natural conditions, and the results showed a significantly greater efficacy for LCTZ, compared with placebo, in reducing the nasal symptom scores and the overall RQLQ scores18.

Fluticasone furoate nasal spray (FFNS) is a novel enhanced-affinity intra-nasal corticosteroid that is administered once daily with the onset of symptom relief as early as 8 h and providing 24 h of symptom relief19. In addition, few systemic adverse effects have been reported, regardless of the duration of use, because of the low systemic bioavailability20. FFNS has been shown to be significantly effective for relieving nasal symptoms, compared with placebo, in adult patients with SAR in double-blind, placebo-controlled studies21. Prior to its approval in Japan in 2010, a Phase III, randomized, double-blind, placebo-controlled clinical study was conducted to compare the efficacy of the once-daily use of FFNS (110 μg) vs the twice-daily use of fluticasone propionate nasal spray (FPNS) (200 μg) for the treatment of adult patients with JC pollinosis22. The results showed that FFNS was effective for improving the nasal symptoms of patients with JC pollinosis and was non-inferior to FPNS.

Although a combination therapy of an AH and an INS is recommended in the Japanese guidelines for the treatment of SAR2, little clinical evidence of the efficacy of such a regimen has been available in Japan to date. Therefore, we assessed the clinical efficacy of the combination therapy with FFNS and LCTZ, compared with LCTZ monotherapy, for suppressing nasal and ocular symptoms under well-controlled conditions in an OHIO Chamber.

## Methods

### Study population

Healthy subjects aged 20–65 years of age who had a history of Japanese cedar pollinosis, of at least 2 years, with moderate-to-severe symptoms and who had a positive CAP-RAST score (class ≥ 2) for Japanese cedar were recruited. Subjects were excluded if they had nasal diseases (nasal polyps and/or deviated nasal septum) or an infectious disease (acute rhinitis, chronic rhinitis, congestive sinusitis, atrophic rhinitis, chronic rhinosinusitis, and flu-associated rhinitis) that would interfere with the evaluation of the efficacy of the drug. Subjects were also excluded if they had a systemic disease, including asthma, hypertension, and diabetes mellitus, or if they had undergone nasal surgery and immunotherapy for the purpose of treating AR. Female subjects who wanted to become pregnant or pregnant women or breast-feeding women were excluded. Subjects who were considered ineligible by the physician in charge were also excluded. The following medications were prohibited throughout the study period: steroid injections, oral or intra-nasal steroids, antihistamines, all LTRAs, tranquilizers, or topical decongestants.

This study was conducted in conformity with the Good Clinical Practice guidelines and the Declaration of Helsinki of 1995 (as revised in Edinburgh in 2000). The study protocol was reviewed and approved by an independent institutional review board at Shinanozaka Clinic (Tokyo, Japan). Informed consent was obtained from all the subjects prior to their participation in the study.

### Study design and protocol

**Figure 1.**
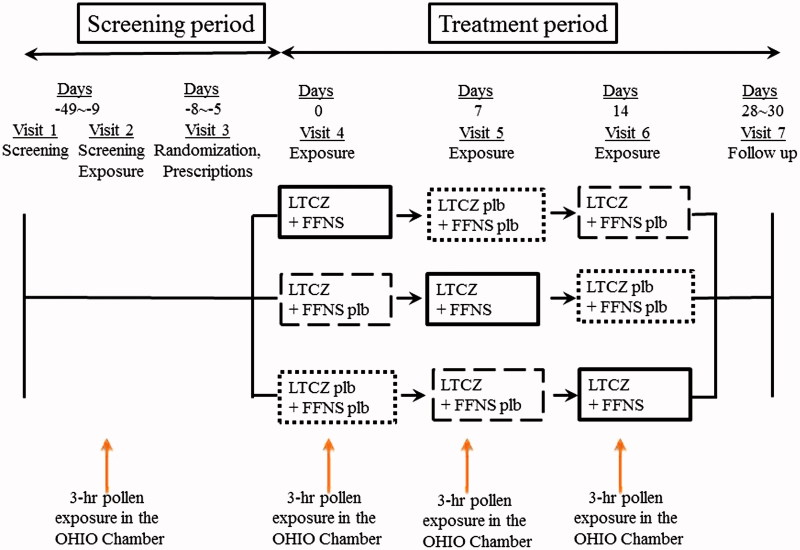
Study protocol. Subjects were randomized to receive either levocetirizine (LCTZ) (5 mg) and fluticasone furoate (FFNS) (55 μg/each nostril), LCTZ (5 mg) and FFNS placebo, or LCTZ placebo and FFNS placebo once before going to bed on the day prior to exposure and after a 1-week washout period.

This was a single-center, randomized, double-blind, placebo-controlled, 3-period cross-over study performed using an OHIO Chamber. The study was conducted outside of the pollen season from October to December in 2011. The study protocol is shown in Figure 1. During the screening visit, which was 2 weeks before the first treatment visit, subjects who met the inclusion criteria and who did not fulfill the exclusion criteria were exposed to pollen in the OHIO Chamber at a concentration of 8000 grains/m^3^ for 3 h.

Eligible subjects were enrolled in the double-blind treatment and were randomized to receive either LCTZ (5 mg) plus FFNS (55 μg/each nostril) (combination group), LCTZ (5 mg) plus FFNS placebo (monotherapy group), or LCTZ placebo plus FFNS placebo (placebo group) once before going to bed on the day prior to exposure and after a 1-week washout period (Figure 1).

The external appearances of the placebos were indistinguishable from the actual drugs.

### JC pollen exposure

Exposure to JC pollen was conducted in an OHIO Chamber that had been set to disperse a constant concentration of JC pollen under a constant humidity (45% ± 5%) and temperature (22°C ± 0.5°C); the chamber was located at the Samoncho Clinic in Tokyo. This facility can accommodate up to 14 subjects in one setting. The concentration of JC pollen and the exposure time were set at 8000 grains/m^3^ (measured every 3 min by laser particle counter) and 3 h, respectively, according to the findings of a previous validation study23.

### Assessment of symptoms

Subjects were instructed to grade and record the severity of their nasal and ocular symptoms (sneezing, rhinorrhea, nasal congestion, itchy nose, and eye itching and tearing) during every 15 min of exposure in the OHIO Chamber and every 8 h on the day after leaving the chamber as well as the next 6 days according to the following 5-point scale: 0 = none (no symptoms); 1 = mild (symptoms present but easily tolerated); 2 = moderate (awareness of symptoms, bothersome but tolerable); 3 = severe (definite awareness of symptoms; difficult to tolerate but does not interfere with activities of daily living); and 4 = very severe (difficult to tolerate and interferes with activities of daily living). This rating system has been validated in the previous study23 and is different from that used in clinical trials in Europe and the US.

The amount of nasal secretions was measured by collecting previously weighed facial tissues every 30 min. The weight difference between the tissues before and after use was considered as the amount of nasal discharge. The number of sneezes was counted and self-reported by each subject every 15 min.

The primary end-points were the cumulative incidence of nasal and ocular symptoms of the subjects during a 3-h exposure to pollen. A score of 1 or more for nasal (sneezing, rhinorrhea, nasal congestion, and itchy nose) and ocular (eye itching and tearing) symptoms was regarded as positive according to the subjects’ self-rated score. The cumulative incidence of nasal or ocular symptoms corresponded to the number of subjects whose nasal or ocular symptoms became positive for the first time prior to the measurement point. The interval from the start of exposure until the occurrence of the first nasal symptom (any of the four nasal symptoms) was designated as the ‘time to occurrence of symptoms’, a second primary end-point. The secondary end-points were the sum of the mean score of the four nasal symptom scores (TNSS) and two ocular symptom scores (TOSS) at each measurement point during exposure and the mean area under the curve (AUC) for individual nasal and ocular symptoms during exposure and for 6 consecutive days after leaving the OHIO chamber. For the objective measurements, changes in the amount of nasal secretions and the number of sneezes during each 1-h period were compared among the treatment groups.

### Statistical analysis

The cumulative incidences of nasal and ocular symptoms of the subjects were plotted for every measurement point after the calculation of the symptom incidence and the 95% confidence interval (CI). Comparisons of primary end-points among the treatment groups were analyzed using the McNemar test. Statistical inference and testing of the differences in the ‘time to occurrence of symptoms’ among the treatment groups were performed using the hazard ratio with the 95% CI estimated using the Cox model with shared frailty after calculating the cumulative incidence and the median time of occurrence at every point using the Kaplan-Meier method.

Comparisons of the TNSS or TOSS among the treatment groups were analyzed using a multi-level model (random effects model) with each score and the treatment methods used as the continuous variable and the dummy variables, respectively. The model was adopted to consider that the measurements were made repeatedly for each subject. The mean areas under the curve (AUCs) for individual nasal symptoms during exposure were also analyzed. The same analysis was applied to compare the amount of nasal discharge, the number of sneezes, and the number of nose blowing events.

All the statistical tests were two-sided, and the level of significance was set at 5%.

### Sample size

The cumulative incidences of symptoms after 3 h of pollen exposure among the subjects treated with FFNS + LCTZ, LCTZ + placebo, or placebo + placebo were expected to be 55%, 80%, and 100%, respectively. Assuming the use of a McNemar test with a two-tailed alpha error of 5%, 40 subjects would be sufficient to detect a difference in the cumulative incidence from 80% (monotherapy) to 55% (combination therapy) with a statistical power of 80%, allowing 3% point mixing to discordant incident categories between different exposure tests. Anticipating a dropout rate of 5%, the subject number was set at 42.

### Safety

Safety was evaluated throughout the study period. All the subjects were asked about their health status and adverse reactions during the study period.

## Results

### Subjects

A total of 107 adult patients with JC pollinosis were recruited and screened; of these patients, 42 eligible subjects were enrolled in the study and were randomized to a treatment group. The demographics of the subjects are shown in Table 1. Two subjects were not able to visit the clinic for a scheduled exposure: one did not take the placebo + placebo because of an upper respiratory infection, and the other did not take the LCTZ + placebo because of bruising after a traffic accident. Therefore, the numbers of subjects in the combination, monotherapy and placebo groups were 42, 41, and 41, respectively.

**Table 1.** Demographic and baseline characteristics.

Gender	
Male, *n* (%)	10 (23.8%)
Female, *n* (%)	32 (76.2%)
Age Mean (SD)	45.1 (8.4)
Duration of SAR Mean (SD)	19.9 (9.2)
Onset of age of SAR Mean (SD)	25.2 (9.4)
RAST score of JC Mean (SD)	3.58 (0.80)

### Cumulative incidence of nasal and ocular symptoms of subjects

The cumulative incidence of nasal and ocular symptoms in the subjects was lowest in the combination group, followed by the monotherapy and the placebo groups, at all the measurement points.

**Figure 2.**
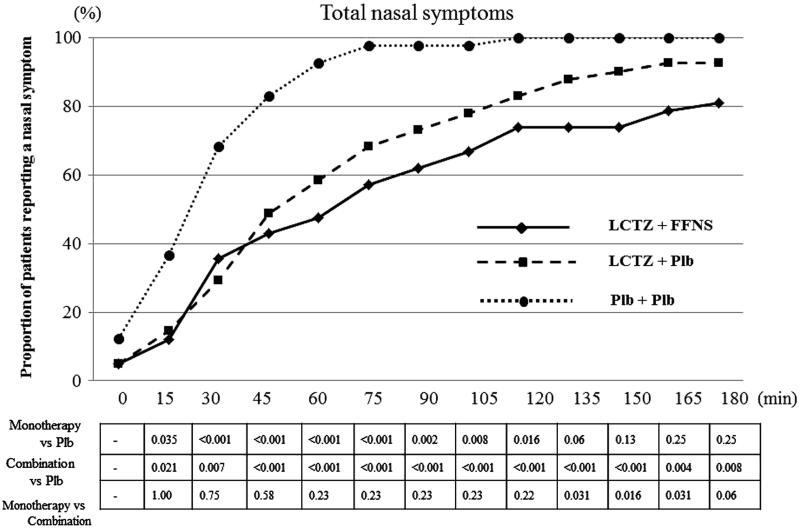
The cumulative incidences for nasal symptoms of the subjects during exposure by time point. All the subjects developed nasal symptoms after 120 min of dispersing pollen in the placebo group. However, three and eight subjects did not develop any nasal symptoms during exposure in the monotherapy and combination groups, respectively. Combination, combination therapy with levocetirizine and fluticasone furoate; Monotherapy, levocetirizine monotherapy.

All the subjects developed nasal symptoms (sneezing, rhinorrhea, nasal congestion, and nasal itching) after 120 min of pollen dispersal in the placebo group. In contrast, three and eight subjects did not develop any nasal symptoms during exposure in the monotherapy and the combination groups, respectively. Compared with the placebo group, the combination groups had significantly lower incidence of total nasal symptoms until the end of exposure (*p* < 0.01 for 30, 165, and 180 min, *p* < 0.001 from 45 to 150 min). A significant difference in the cumulative incidence of total nasal symptoms was observed between the monotherapy and the combination groups during the latter half of the exposure (from 135 min to 165 min) (*p* = 0.031, 0.016, and 0.031 for 135, 150 and 165 min, respectively) (Figure 2).

### Time until occurrence of symptoms

**Figure 3.**
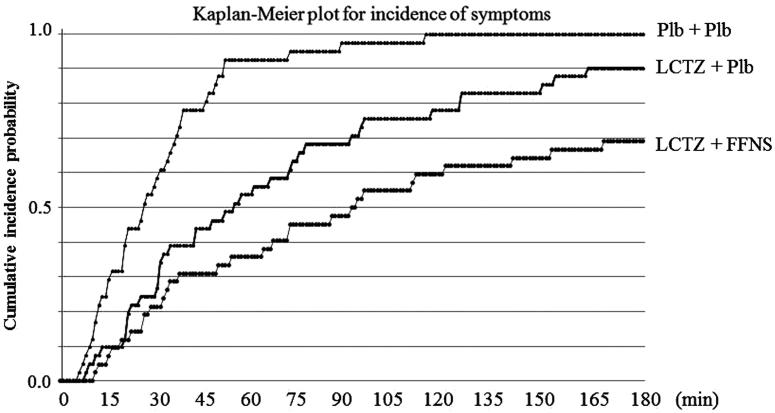
Kaplan-Meier plot for incidence of symptoms. LTCZ, levocetirizine; FFNS, fluticasone furoate nasal spray; Plb, placebo.

The median times until the occurrence of any one of four nasal symptoms (95% CI) in the placebo, monotherapy, and combination groups were 26 min (20–34 min), 54 min (31–74 min), and 90 min (53–140 min), respectively, using the Kaplan-Meier method (Table 2 and Figure 3). Table 2 shows the hazard ratio (95% CI) between each of the treatment groups. These results indicated that the suppression of nasal symptoms was most effective in the combination group, followed by the monotherapy and placebo groups.

**Table 2.** Median time until the occurrence of nasal symptoms and Hazard ratio between treatment groups.

	Median time	(95% CI)	Hazard ratio*	(95% CI)	*p*
Plb + Plb	26 min	(20, 34 min)	0.290	(0.174, 0.438)	<0.001
LCTZ + Plb	54 min	(31, 74 min)	0.136	(0.075, 0.245)	<0.001
LCTZ + FFNS	90 min	(53, 140 min)	0.468	(0.279, 0.785)	0.004

*Hazard ratios with the 95% CIs were estimated by Cox model with shared frailty. LTCZ, levocetirizine; FFNS, fluticasone furoate nasal spray, Plb, placebo; CI, confidence interval.

### Total nasal symptom scores (TNSSs) and AUCs for nasal and ocular symptoms

**Figure 4.**
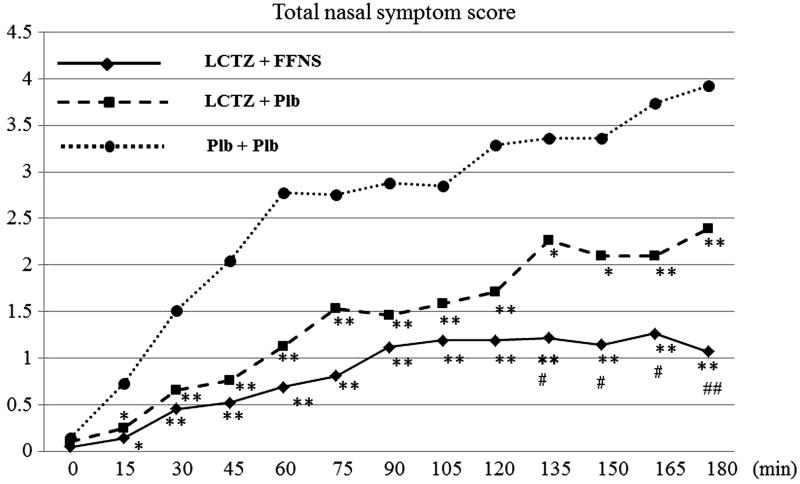
Time course of the total nasal symptom scores (TNSSs). ** vs Placebo < 0.001, * vs Placebo < 0.01, ## vs Monotherapy < 0.001, # vs Monotherapy < 0.05. LTCZ, levocetirizine; FFNS, fluticasone furoate nasal spray; Plb, placebo.

The TNSSs for both the combination and monotherapy groups were significantly lower than that in the placebo group from 15 min after the start of exposure until the end of exposure. Furthermore, the TNSS scores in the combination group were lower than those in the monotherapy group, with a significant difference apparent from 135 min after the start of exposure (*p* < 0.05) (Figure 4).

The AUCs for rhinorrhea, sneezing, nasal itching, and nasal congestion in both the combination and the monotherapy groups were significantly lower than those in the placebo group (all *p* < 0.001 except for nasal congestion in monotherapy vs placebo, for which *p* = 0.002). Comparisons between the combination and monotherapy groups showed a significant difference in the AUCs for rhinorrhea (*p* = 0.01) and nasal congestion (*p* = 0.02), but no significant differences were found for sneezing and nasal itching (*p* = 0.07 and *p* = 0.22, respectively) (Table 3).

**Table 3.** Comparisons of AUCs for individual nasal and ocular symptoms.

	AUC (SD)	Difference vs placebo (95% CI)	*p*-value	Difference vs monotherapy (95% CI)	*p*-value
Rhinorrhea				
Combination	2.35 (3.26)	−6.69 (−8.45, −4.94)	<0.001	−2.24 (−3.99, −0.48)	0.01
Monotherapy	4.61 (4.37)	−4.46 (−6.22, −2.69)	<0.001		
Placebo	9.05 (3.26)				
Sneeze					
Combination	1.23 (2.29)	−3.80 (−4.79, −2.81)	<0.001	−0.91 (−1.90, 0.08)	0.07
Monotherapy	2.15 (2.48)	−2.89 (−3.89, −1.89)	<0.001		
Placebo	5.02 (3.49)				
Nasal congestion					
Combination	3.14 (5.53)	−5.14 (−6.98, −3.29)	<0.001	−2.21 (−4.06, −0.36)	0.02
Monotherapy	5.39 (5.48)	−2.92 (−4.79, −1.06)	0.002		
Placebo	8.34 (6.26)				
Nasal itching					
Combination	3.58 (4.78)	−5.38 (−7.17, −3.59)	<0.001	−1.11 (−2.90, 0.68)	0.22
Monotherapy	4.63 (5.29)	−4.27 (−6.07, −2.46)	<0.001		
Placebo	8.94 (5.98)				
Total nasal symptoms					
Combination	10.30 (12.53)	−21.02 (−26.43, −15.62)	<0.001	−6.46 (−11.86, −1.06)	0.02
Monotherapy	16.78 (14.01)	−14.56 (−20.00, −9.12)	<0.001		
Placebo	31.35 (16.21)				
Eye itching					
Combination	1.76 (3.51)	−2.12 (−3.49, −0.76)	<0.01	−0.20 (−1.57, 1.17)	0.77
Monotherapy	1.94 (3.72)	−1.92 (−3.30, −0.55)	0.01		
Placebo	3.90 (4.49)				
Tearring					
Combination	0.46 (1.74)	−1.55 (−2.21, −0.88)	<0.001	−0.39 (−1.05, 0.28)	0.25
Monotherapy	0.80 (2.46)	−1.16 (−1.83, −0.49)	<0.001		
Placebo	2.02 (3.87)				
Total ocular symptom					
Combination	2.23 (4.38)	−3.67 (−5.36, −1.98)	<0.001	−0.59 (−2.28, 1.10)	0.50
Monotherapy	2.74 (5.23)	−3.08 (−4.78, −1.38)	<0.001		
Placebo	5.93 (7.19)				

AUC, area under curve; Combination, combination therapy with levocetirizine and fluticasone furoate; Monotherapy, levocetirizine monotherapy.

The AUCs for the total ocular symptoms scores in the combination and monotherapy groups were significantly lower than that in the placebo group (*p* < 0.001). However, no significant differences in the AUCs for ocular itching and tearing were observed between the active treatment groups (*p* = 0.77 and *p* = 0.25, respectively) (Table 3).

**Figure 5.**
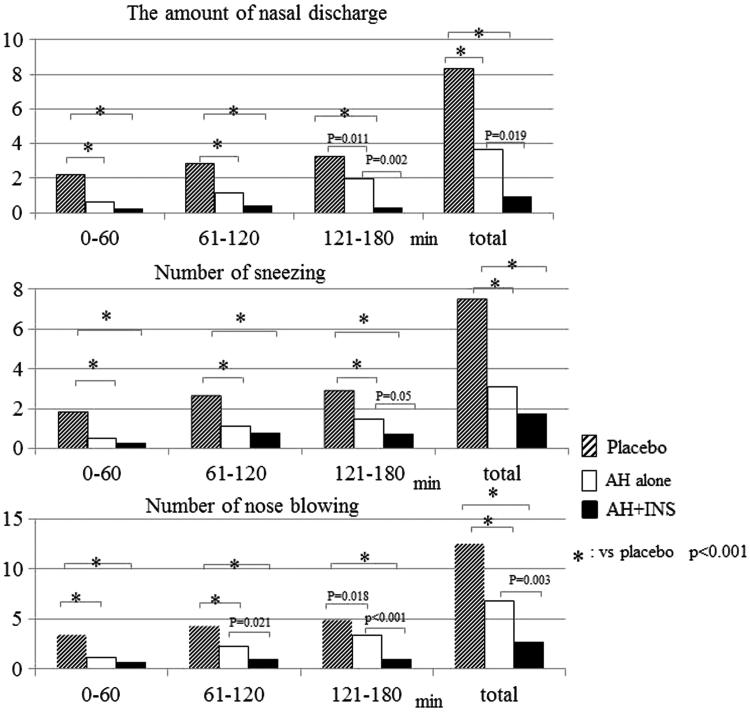
The results of the amount of nasal discharge, number of sneezing, and nose blowing. LTCZ, levocetirizine; FFNS, fluticasone furoate nasal spray; Plb, placebo.

Both the combination and the monotherapy groups had significantly lower amounts of nasal secretions than the placebo group (*p* < 0.001) over the exposure period, and the combination group showed a significantly lower value than the monotherapy group during the last 1 h of exposure (*p* = 0.002). Similar trends were observed for the comparisons of the number of sneezes and nose blowing events between the treatment groups (Figure 5). Although no significant difference in the number of sneezes was observed between the active treatment groups, both active treatment groups had a significantly lower number of sneezes than the placebo group (*p* < 0.001). Similar results were obtained for a comparison of the number of nose blowing events, which is troublesome for SAR patients and may deteriorate patients’ QoL. The combination group had a lower number of nose blowing events than the monotherapy group at 1 h after the start of exposure, and a significant difference was observed overall (*p* = 0.003).

### AUCs for nasal and ocular symptoms after leaving the OHIO chamber (Table 4)

After returning home on the day of exposure (day 0), the AUCs for sneezing and rhinorrhea in both of the active treatment groups were significantly lower than that in the placebo group (*p* < 0.001 and *p* < 0.003, respectively), and the difference between the combination and monotherapy groups was also statistically significant (*p* < 0.001), while the AUC for nasal congestion was significantly lower for the combination group, compared with the monotherapy and placebo groups (*p* < 0.001). On the following day (day 1), the AUCs for all three symptoms in the combination group were significantly lower than those in the monotherapy group (*p* ≤ 0.001) and the placebo group (*p* < 0.001). No significant difference was observed between the monotherapy and the placebo groups. On the second day after exposure (day 2), no significant differences in the AUC for nasal congestion were found among the three groups, while significant differences in rhinorrhea, sneezing, and nasal itching were observed between the combination and monotherapy groups (*p* = 0.006, 0.001, and 0.009, respectively). After the third day of exposure, no significant differences in nasal symptoms were observed among the three groups.

**Table 4.** AUCs for individual nasal symptom after leaving the OHIO chamber.

	Treatment group			Difference		
	Combination Mean (SD)	Monotherapy Mean (SD)	Placebo Mean (SD)	Com vs Plb Mean (95% CI) *p*-value	Mono vs Plb Mean (95% CI) *p*-value	Com vs Mono Mean (95% CI) *p*-value
*Rhinorrhea*						
Day 0	0.19 (0.16)	0.34 (0.25)	0.47 (0.27)	−0.27 (−0.36, −0.19)	−0.13 (−0.21, −0.04)	−0.15 (−0.23, −0.07)
				<0.001	0.003	<0.001
Day 1	0.29 (0.27)	0.63 (0.54)	0.64 (0.44)	−0.35 (−0.50, −020)	−0.01 (−0.16, 0.14)	−0.34 (−0.49, −0.19)
				<0.001	0.88	<0.001
Day 2	0.31 (0.34)	0.49 (0.40)	0.42 (0.41)	−0.11(−0.23, 0.02)	−0.06 (−0.06, 0.19)	−0.17 (−0.30, −0.005)
				0.09	0.31	0.006
*Sneeze*						
Day 0	0.17 (0.14)	0.30 (0.20)	0.42 (0.24)	−0.26 (−0.33, −0.18)	−0.12 (−0.19, −0.04)	−0.14 (−0.22, −0.06)
				<0.001	0.003	<0.001
Day 1	0.26 (0.25)	0.57 (0.45)	0.59 (0.42)	−1.36 (−1.98, −0.74)	−0.02 (−0.17, 0.13)	−0.31 (−0.46, −0.16)
				<0.001	0.81	<0.001
Day 2	0.27 (0.28)	0.42 (0.33)	0.39 (0.32)	−0.13 (−0.23, −0.03)	0.03 (−0.07, 0.13)	−0.16 (−0.26, −0.06)
				0.011	0.52	0.001
*Nasal congestion*						
Day 0	0.10 (0.14)	0.26 (0.26)	0.34 (0.30)	−0.24 (−0.32, −0.16)	−0.07 (−0.16, 0.01)	−0.17 (−0.25, −0.08)
				<0.001	0.09	<0.001
Day 1	0.12 (0.26)	0.34 (0.44)	0.41 (0.45)	−0.28 (−0.41, −0.16)	−0.05 (−0.18, 0.08)	−0.23 (−0.36, −0.10)
				<0.001	0.42	<0.001
Day 2	0.12 (0.25)	0.22 (0.38)	0.20 (0.30)	−0.08 (−0.18, 0.03)	0.01 (−0.09, 0.12)	−0.09 (−0.20, 0.01)
				0.14	0.79	0.08
*Nasal itching*						
Day 0	0.09 (0.13)	0.18 (0.22)	0.33 (0.25)	−0.24 (−0.31, −0.17)	−0.15 (−0.22, −0.07)	−0.09 (−0.17, −0.02)
				<0.001	<0.001	0.01
Day 1	0.10 (0.22)	0.31 (0.50)	0.44 (0.43)	−0.35 (−0.49, −0.22)	−0.12 (−0.26, 0.01)	−0.23 (−0.37, −0.09)
				<0.001	0.08	0.001
Day 2	0.12 (0.22)	0.26 (0.45)	0.24 (0.32)	−0.13 (−0.25, −0.11)	0.03 (−0.09, 0.14)	−0.16 (−0.27, −0.04)
				0.031	0.66	0.009

AUC, area under curve; Combination, combination therapy with levocetirizine and fluticasone furoate; Monotherapy, levocetirizine monotherapy; Plb, placebo.

Similar trends were observed in the scores for ocular symptoms among the treatment groups (data not shown). No significant differences between the combination and the monotherapy group were observed during the observation period after leaving the OHIO chamber.

### Safety

No serious adverse events were observed throughout this study. Seven adverse events were documented in five subjects (common cold [*n* = 2], headache [*n* = 1], dog bite [*n* = 1], conjunctivitis [*n* = 1], bruising [*n* = 1], and pharyngitis [*n* = 1]). All the adverse events were mild and were regarded as being unrelated to the test drugs or study procedure.

## Discussion

This is the first comparative study to investigate the efficacy of combination therapy with FFNS plus LCTZ and LCTZ monotherapy in a controlled JC pollen exposure setting while assessing the cumulative incidence of nasal symptoms and the time of occurrence as primary end-points. The evaluation of onset of symptoms was thought to be clinically significant as well as evaluation of nasal and ocular symptoms. Our results clearly demonstrated that combination therapy with FFNS and LCTZ, administered on the night prior to the pollen exposure, suppressed the symptoms induced by JC pollen exposure in the OHIO chamber, compared with LCTZ monotherapy or placebo, and significantly delayed the onset of symptoms. All the subjects in the placebo group developed nasal symptoms, while three and eight subjects in the monotherapy and combination groups, respectively, did not show any nasal symptoms during exposure. The scores for nasal and ocular symptoms in both treatment groups were significantly lower than those in the placebo group. For individual symptoms, significant differences in the scores for rhinorrhea and nasal congestion, but not for sneezing and the two ocular symptoms, were observed between the combination and monotherapy groups.

Second generation AHs are usually effective against sneezing and rhinorrhea through H_1_ receptor blockade but are less effective against nasal congestion, which is induced by mediators other than histamine5,24. LCTZ is a potent anti-allergic drug, classified as a second-generation antihistamine, with an early onset of action and a long duration of effects on the symptoms of AR25. Some studies have shown that LCTZ improves not only sneezing and rhinorrhea, but also nasal congestion in patients with AR when analyzed using subjective scores or objective measurements (rhinomanometry)26–28. In this study, the individual nasal scores (sneezing, rhinorrhea, nasal congestion, and nasal itching) measured during the exposure period were significantly lower in the LCTZ monotherapy group compared with the placebo group. In terms of the cumulative incidence of nasal symptoms, however, the suppression of nasal congestion in the monotherapy group was not as prominent as the suppressions of the other nasal symptoms. The present result showed that combination therapy with FFNS and LCTZ was superior to LCTZ monotherapy for the suppression of nasal congestion. Interestingly, the scores for non-nasal symptoms, such as eye itching and tearing, were significantly lower in the monotherapy group than in the placebo group.

Previously, five randomized, double-blind, placebo-controlled studies investigated the efficacy of LCTZ and other active antihistamines in ragweed-sensitive patients subjected to environmental pollen exposure facilities28–32. Of these, two studies were conducted in the Vienna Challenge Chamber (VCC) in a cross-over design and compared the efficacy of levocetirizine, loratadine/fexofenadine, and placebo28,29. Two other comparative studies were conducted in an environmental exposure unit (EEU) by Day *et al*.30,31 and in an environmental exposure chamber (EEC) by Patel and Patel 32 to evaluate the efficacy of levocetirizine with desloratadine or montelukast as a control medicine in studies with a parallel-group design. These studies demonstrated that LCTZ was significantly more effective than other active AHs and placebo in reducing AR symptoms induced by pollen exposure and that LCTZ had a faster onset of action (0.75–2.5 h). The present study, despite differences in the study design, confirmed the efficacy of LCTZ for suppressing the development of AR symptoms, compared with placebo, even when administered in advance prior to pollen exposure.

INSs are potent drugs that are usually effective not only for nasal congestion, rhinorrhea, sneezing, and nasal itching, but also for ocular symptoms, such as ocular itching and tearing, in adults and children with SAR and perennial allergic rhinitis (PAR); these effects arise through different kinds of pharmacologic actions via the binding of corticosteroids to intracellular glucocorticoid receptors, resulting in strong anti-inflammatory effects33. Therefore, INSs are recommended for the treatment of moderate or severe symptoms of AR.

FFNS is a recently developed intra-nasal corticosteroid that is administered once daily and has a potent anti-inflammatory effect. FFNS has few systemic adverse effects due to its low bioavailability. Its significant efficacy in the relief of nasal symptoms, compared with placebo, has been demonstrated in adult and adolescent patients with SAR in double-blind, placebo-controlled studies19,34. An exposure chamber trial conducted by Zieglmayer *et al*.35 in the VCC evaluated the efficacy of a 1-week treatment with high-dose (200 µg) FFNS in grass-pollen sensitized patients and showed a significant reduction in AR symptoms and a long duration of effects. However, no previous study has investigated the efficacy of an optimal dose of FFNS administered in a pollen exposure facility.

We did not examine FFNS monotherapy in this study in consideration of the actual circumstances in Japan, where the prescription rate for INSs by GPs and the use of INSs in patients with pollinosis are both relatively low14,16. The addition of FFNS to LCTZ, however, demonstrated a significant suppression and delay in the development of nasal symptoms, compared with LCTZ monotherapy, in the present study, indirectly but clearly indicating the potent efficacy of FFNS. These results seem to be consistent with a double-blind, placebo-controlled, parallel-group EEU study reported by Day e*t al*.36, who investigated the onset of action and the efficacy of the single use of two doses of intranasal budesonide aqueous nasal spray (BANS), an INS for the treatment of AR, in ragweed-sensitized subjects to whom the test drugs were administered after the development of symptoms36. They found that BANS had a faster onset of action and a larger impact on the reduction of nasal symptoms than placebo.

Oral anti-histamines and intra-nasal steroids have different mechanisms of action on allergic reactions. The former exert a rapid reduction in early-phase AR symptoms, such as sneezing, rhinorrhea, and nasal itching, through their effects on histamine5, while the latter have inhibitory effects mainly on late-phase AR reactions through the reduction of inflammatory cell recruitment and inflammatory mediator release33. Therefore, a combination therapy involving both an AH and an INS might have combined effects on allergen-induced symptoms and seems to be of clinical benefit. However, previous studies that investigated the clinical efficacy of a combination therapy with INS and AH compared with INS or AH administered alone for the treatment of SAR showed that combination therapy was non-inferior and not superior to INS monotherapy, with no clinical benefit7–11. Despite these clinical study results, conflicting data exist regarding the prescription rates for medications for the treatment of AR. The prescription rates for AH, a combination of AH and INS, and INS alone were 42.8%, 31.4%, and 14.8%, respectively, in a pan-European study13 and 31.5%, 42.3%, and 21.5%, respectively, in an American study12. Interestingly, the prescription rate for a combination of AH and INS was much higher (66%) in a survey in Spain37. Similarly, the rate of single-use of INS treatments in Japan was relatively low according to a questionnaire survey conducted over the internet14, despite the large number of JC pollinosis patients with moderate/severe symptoms.

Our results showed that combination therapy with FFNS and LCTZ provided significant suppression of nasal and ocular symptoms from the start to the end of pollen exposure in terms of the cumulative incidence of symptoms and the symptom scores, compared with placebo. Nasal symptoms, especially nasal congestion, were more efficiently suppressed during the latter half of the study period in the combination group, compared with the monotherapy group. Of note, significantly lower nasal scores were observed in the combination group at home on the day of and on the day after exposure, compared with the monotherapy and placebo groups. The combination therapy, however, did not result in a more efficacious suppression of ocular symptoms, compared with the monotherapy, the long-term use of FFNS might be necessary to obtain clinical efficacy for the suppression of ocular symptoms, as indicated in the previous clinical studies21,34.

There are some limitations to the present study. Usually INSs need several days of administration to achieve their full anti-inflammatory efficacy, while only one application of FFNS was used in this study in consideration of Japanese circumstances. Antigen challenge studies using exposure chambers are conducted under well-controlled conditions, but the restricted area places some limitations on the subjects’ actions. And the subjects are exposed to pollen allergens for a short period of time, while in the real world pollen exposure is much longer and variable. Although these circumstances differ from those experienced during real life, a recent study demonstrated that symptoms elicited in such a chamber are similar to those observed during pollen season under natural conditions38. Thus, this study design and the study results are considered to be valid for clinical settings.

## Conclusion

The present study demonstrated that the combination therapy of FFNS and LCTZ, in the OHIO chamber, was more efficacious for suppressing or preventing nasal symptoms than LCTZ monotherapy, which is the mainstay of treatment for JC pollinosis. Although the present study was conducted in an artificial pollen exposure chamber outside the pollen season, the results provide the first evidence of a beneficial effect for the treatment of SAR in Japan.

## Transparency

### Declaration of financial/other interest

KO has received a research grant and lecture fees from GlaxoSmithKline. ST has received consulting fees from a NPO which is funded by GlaxoSmithKline. The rest of the authors have no competing interest to declare.
